# Diagnostic Challenges and Surgical Management of Ovarian Hydatid Cyst: A Case Report From an Endemic Region and Literature Review

**DOI:** 10.1002/ccr3.72113

**Published:** 2026-03-05

**Authors:** Fatemeh Asadi Kohbad, Razieh Akbari, Maryam Forouzin, Fatemeh Nili, Leila Asadi, Marjan Ghaemi

**Affiliations:** ^1^ Vali‐E‐Asr Reproductive Health Research Center, Family Health Research Institute Tehran University of Medical Science Tehran Iran; ^2^ School of Medicine Urmia University of Medical Sciences Urmia Iran; ^3^ Department of Pathology Tehran University of Medical Sciences Tehran Iran

**Keywords:** cyst, hydatid, hydatid cyst, ovarian

## Abstract

Ovarian hydatid cysts are an infrequent presentation of the zoonotic condition caused by *Echinococcus granulosus* tapeworms. These cysts can easily be mistaken for benign or malignant ovarian neoplasms, posing a diagnostic challenge. This case report describes managing a case of ovarian hydatid cyst in an endemic region. A 50‐year‐old woman, who had previously undergone surgery for a liver hydatid cyst, presented with abdominal pain and amenorrhea. Upon further investigations, it was revealed that a multilocular cystic lesion was in her left ovary through ultrasonography and computed tomography. Tumor markers were within normal limits. Intraoperative evaluation confirmed the diagnosis of a hydatid cyst, leading to a decision for a total abdominal hysterectomy with bilateral salpingo‐oophorectomy. The surgery was performed to remove the cyst safely. Following the procedure, the patient received anthelmintic therapy with albendazole. The outpatient follow‐up was focused on monitoring the patient's recovery and response to treatment. In conclusion, ovarian hydatid cysts are a rare entity that can mimic ovarian neoplasms, particularly in endemic regions. A high index of suspicion, along with appropriate imaging and histopathological examination, is crucial for accurate preoperative diagnosis. Surgical management, combined with perioperative anthelmintic therapy, is the mainstay of treatment to minimize the risk of recurrence and complications. Clinicians should be vigilant in considering hydatid disease as a differential diagnosis for cystic ovarian masses in endemic regions, even in the absence of a known history of the disease.

## Key Clinical Message

Ovarian hydatid cysts, rare yet resembling ovarian neoplasms, require careful differential diagnosis in endemic areas. Preoperative suspicion, imaging, and histopathology are essential. Surgical treatment, coupled with perioperative anthelmintic therapy, remains the cornerstone to prevent recurrence and complications. Clinicians must consider hydatid disease, even without a prior history, when evaluating cystic ovarian masses.

## Graphical abstract

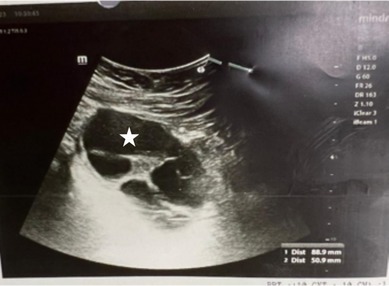

## 1 Background

Cystic echinococcosis can be a life‐threatening helminthic disease, which *Echinococcus granulosus* induces. It occurs worldwide but is most common in some parts of Eurasia (especially the Mediterranean region), north and east Africa, Australia, and South America [1]. Mortality of cystic echinococcosis is considerable in endemic areas. Studies report mortality rates ranging from 1.29% to 1.94% [2]. Cysts are almost always hepatic and/or found in the lung, although symptomatic cysts have been observed in several organs with much less incidence including the peritoneum, Spleen, kidney, heart muscle, vascular system (segmental or general); muscular tissue, brain, and bone [3]. The ovary is the most common location for hydatid cysts within the female genital tract, but overall, this occurrence is infrequent (less than 1% of cases). These cysts typically present in a manner that resembles a malignant tumor [4]. In this case, we represent a patient with an ovary hydatid cyst.

## 2 Case Presentation

A 50‐year‐old woman G2P2, from Karaj in Tehran State, Iran, was hospitalized after experiencing a short instance of abdominal pain in the hypogastric area, particularly in the left lower quadrant (LLQ). The patient said she had been amenorrheic for 8 months and mentioned that she had not had any digestive or urinary issues during this period.

She had surgery for a liver hydatid cyst 3 years ago, but she doesn't remember the specifics. There are no medical records of her previous treatment. Upon admission, the patient was not taking any medication.

## 3 Imaging and Diagnosis

**FIGURE 1:**
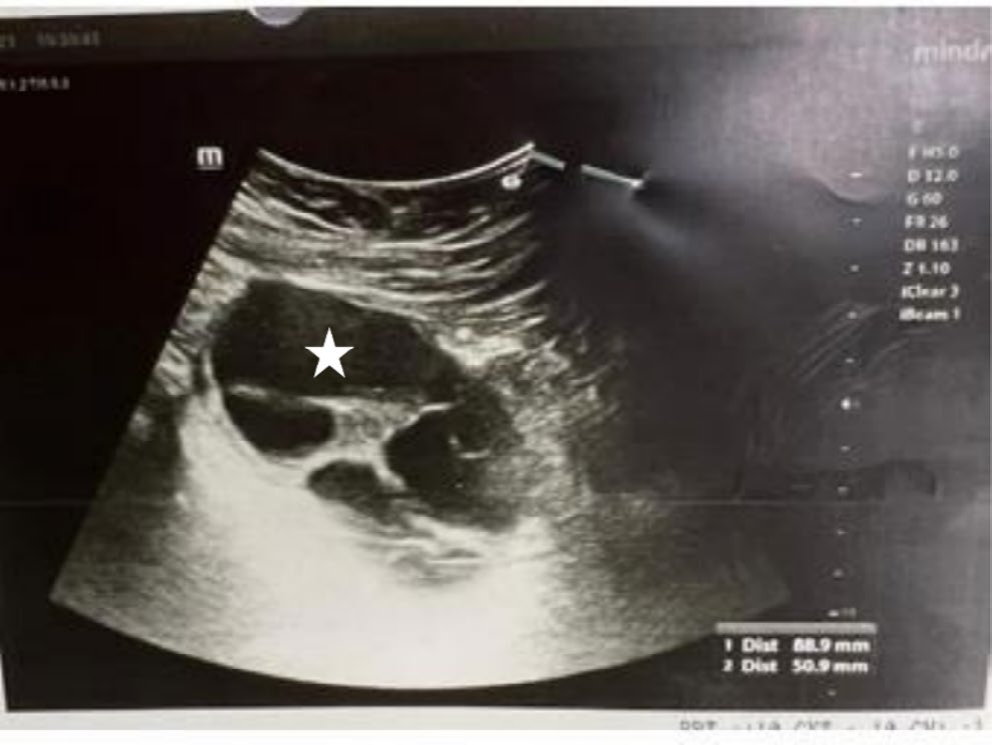
Ultrasound imaging demonstrating a multilocular cystic lesion in the left ovary, characterized by internal echogenic septa, prompting further evaluation to assess potential underlying conditions.

A sonography was requested (Figure 1), which yielded the following findings:

On the imaging examination, the liver was normal in size and echogenic, with no visible hydatid cysts. The portal and hepatic veins were normal. The gallbladder was not seen in its anatomical location (history of cholecystectomy). The intrahepatic and extrahepatic bile ducts had a normal diameter (CBD: 5 mm, PV: 9 mm). No lymphadenopathy was evident in the para‐aortic areas. The spleen was normal in size and echogenicity. The left ovary showed a multilocular cystic lesion measuring 50 × 89 mm. The right ovary was normal in size and echogenicity. There was no free fluid in the pelvis or abdomen. The bladder wall thickness was normal, with no stones or parietal space‐occupying lesions. The radiologist suggested a consultation with a pathologist for tumor markers. On investigation, the tumor markers were within normal limits, and the Pap smear was negative for intraepithelial lesion or malignancy (NILM). There was no leukocytosis, and the inflammatory tests were negative. Following abdominal and pelvic computed tomography (CT) with intravenous and oral contrast, no pathological findings, except for the previous cholecystectomy, were observed. Given the patient's age and the imaging designated, the presence of an 89‐mm cyst in the ovary, accompanied by constant pain, was observed, and the patient became a candidate for surgery.

## 4 Surgery

**FIGURE 2:**
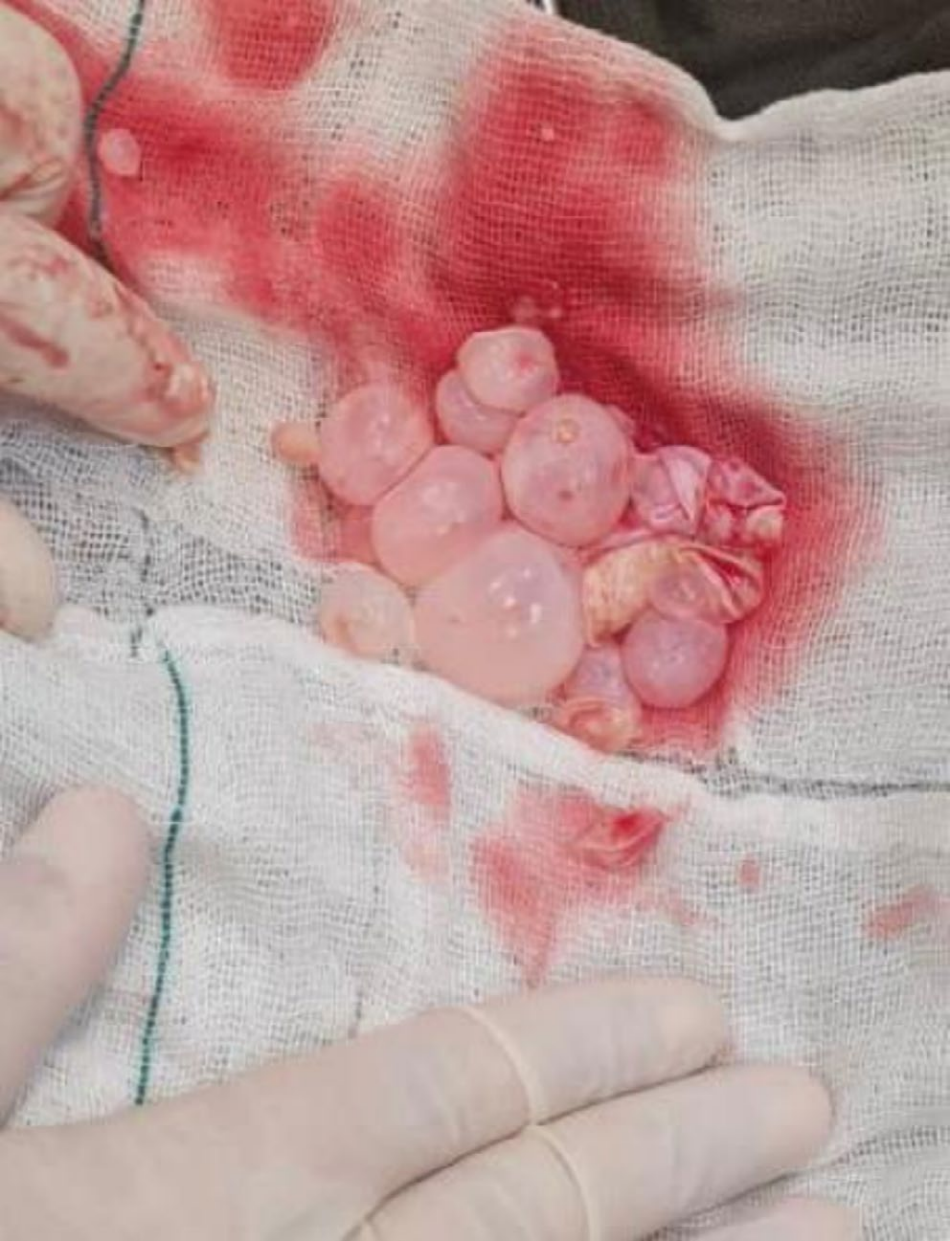
Extracted cysts from the patient with intact membrane.

A bilateral salpingo‐oophorectomy (TAH BSO) was performed on the patient considering her age and surgical risks. This approach minimizes the risk of cyst contents spilling, which is critical to prevent infection recurrence. During the procedure, a sterile gauze was used to avoid contamination, and the cysts were carefully removed and sent to the pathologist in a sterile container. During the surgery, daughter cysts and additional evidence consistent with hydatid cysts were identified, supporting the diagnosis. Postoperatively, an infectious disease consultation was requested to assess potential complications that did not reveal any significant findings that would require further intervention. Following the surgical procedure, the patient was discharged with a medical régime that involved albendazole 400 mg administered every 12 h. She was advised to attend follow‐up appointments at the infectious disease clinic as an outpatient to monitor her condition and ensure proper management (Figure 2).

## 5 Pathology Findings

**FIGURE 3:**
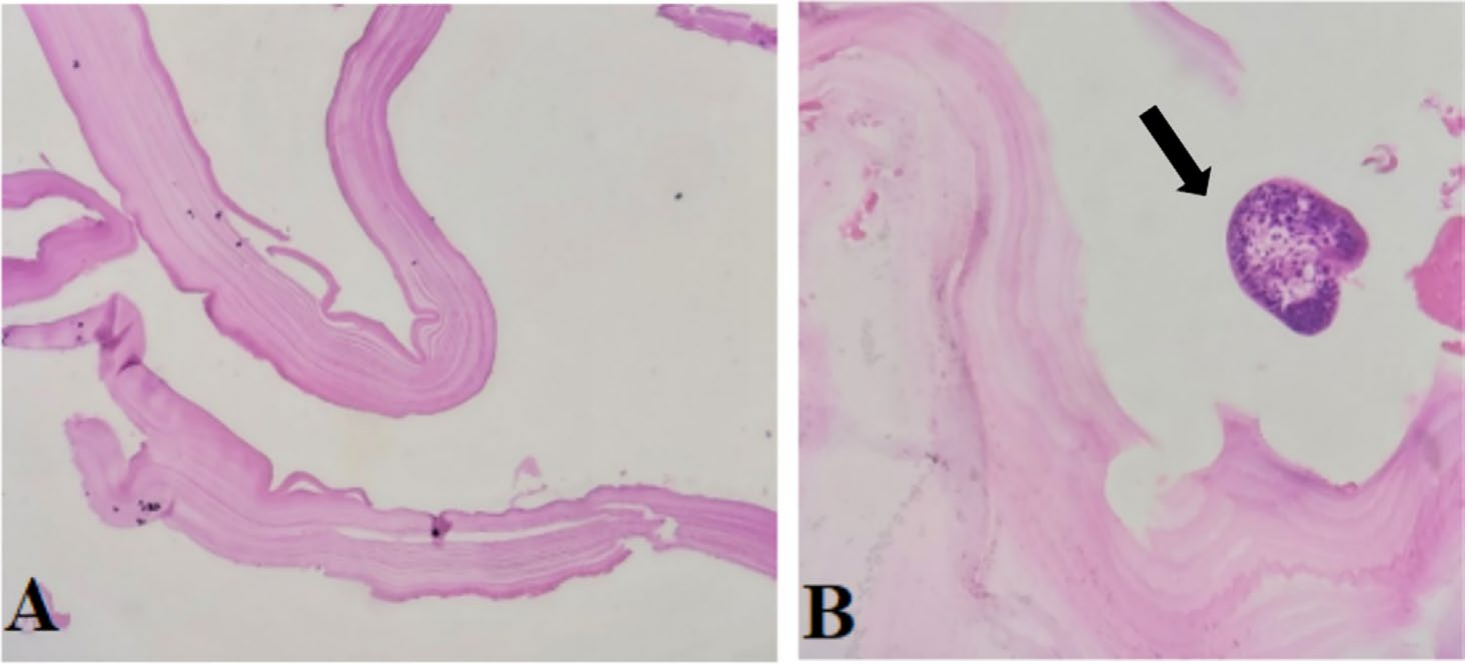
(A) The microscopic examination reveals a cyst wall structure. The outer acellular laminated membrane is observed, showing a characteristic layered appearance: (B) image illustrates the cyst wall, with protoscolices budding from the wall.

The pathology samples were examined using hematoxylin and rosin (H&E) staining, and the results, presented in the following images at 20× magnification (Figure 3).

## 6 Literature Review

In the following section, all similar reports of an ovarian hydatid cyst and the approaches reported are summarized in Table 1.

**TABLE 1:** Summary of similar case reports about ovarian hydatid cyst.

Author	Year	Diagnosis	Treatment	Follow up
L. Cattorini et al. [1].	2011	Clinical examination revealed pain and abdominal distention, attributed to a large hypogastric incisional hernia. An abdominal CT scan reported: A hepatic hydatid cyst (45 mm × 41 mm) in segment 4 of the liver. A multiloculated cyst (68 mm × 62 mm) is situated in the pouch of Douglas.	The patient underwent surgical intervention, which included the following: Opening the hypogastric hernial sac. Lysis of uterine‐vaginal adhesions. Drainage and marsupialization of the cyst (considered an echinococcal cyst) after prolonged viscerolysis. Hysterectomy due to senile atrophic uterus with calcific fibroma.	Post‐operative care involved ensuring the patient recovered without complications and discharging her on the 11th postoperative day. Anticipated follow‐up might include anti‐helminthic therapy to reduce the chance of recurrence, as well as regular imaging or clinical evaluations to monitor for any signs of cyst recurrence or complications.
A.A. Mohammad et al. [5].	2021	lower abdominal pain and frequent urination. The Pain was poorly localized. Ultrasound revealed a cystic left adnexal lesion measuring 56 mm by 39 mm, unilocular and containing fluid.	laparoscopic surgery included the identification of a hydatid cyst in the left ovary. Injection of chlorhexidine around the cyst, followed by aspiration. Extraction of the hydatid cyst using a retrieval bag.	Postoperative care involved a 2‐day hospital admission. Anthelmintic medications were prescribed for 3 months following the surgery to prevent recurrence. A follow‐up ultrasound was scheduled 6 months post‐surgery, showing normal findings.
M.K. Maharlooei et al. [6].	2009	Abdominal pain in the left lower quadrant, primarily at night. Vaginal spotting for several days each month for the past 3 months. Left adnexal mass on bimanual examination—second‐grade uterine prolapse with cystocele and rectocele. CT scan confirmed a multivesiculated cystic mass with peripheral enhancement, raising suspicion for a malignant process. Preoperative tumor marker CA‐125 was elevated at 50 U/mL. The preoperative diagnosis was an ovarian tumor.	The patient underwent laparotomy, during which multiple cysts resembling hydatid cysts were identified in the left ovary. A total abdominal hysterectomy and left salpingo‐oophorectomy were performed.	Pathological examination confirmed the diagnosis of hydatid cysts with no signs of malignancy. Counter‐current immunoelectrophoresis for the detection of hydatid cysts was strongly positive. Follow‐up would typically include monitoring for recurrence and managing any complications related to the surgery.

B. Geramizadeh [7] did a review on unusual hydatid cysts in Iran. In the review, 9 of them were reported as ovarian hydatid cysts, and the preferred method of diagnosis was reported to be ultrasonography, CT scan, and MRI, all of which are much more sensitive than immunologic tests.

## 7 Discussion

Hydatid disease is an exclusive parasitic condition that is endemic in many parts of the world. This parasitic disease is an important public health concern in Iran, an endemic country [8]. Unilocular cysts caused by *Echinococcus granulosus* and multilocular cysts caused by *E. multilocularis* are considered important human diseases due to the negative effect they have on disability‐adjusted life years (DALYs) and economic loss [9]. The clinical manifestations of hydatid cysts in most parts of the body are often too nonspecific to make a diagnosis based only on signs and symptoms before surgical intervention [7]. In a review aimed at assessing the occurrence of hydatid cysts in unusual places endemic to Iran, it was found that according to the literature [7], there have been 9 published cases of ovarian hydatid cysts from Iran.

The initial phase of primary infection is always asymptomatic, with small cysts potentially remaining symptomless for many years or even indefinitely. Symptoms may occur if the cysts rupture or apply pressure, leading to complications like urinary issues or life‐threatening anaphylactic reactions [10]. The nonspecific clinical presentations of the disease make a detailed preoperative diagnosis challenging [1]. Symptomatic examples of the condition may become apparent with characteristic features, but not limited to, abdominal pain, typically localized in the right upper quadrant, jaundice, nausea, vomiting, and abnormalities in liver function tests [11].

The treatment approach for cystic echinococcosis involves the use of anthelmintic drugs both before and after surgery to help decrease the recurrence rate [5]. It is noteworthy that while anthelmintic drugs can be used before surgery in selected cases, the cure rate with medications alone is low, and they are also indicated after surgery to reduce the recurrence rate [5]. Surgical intervention is typically necessary, especially for those at risk of rupture, infected cysts, and those in critical anatomical locations with a significant mass effect. During surgery, it is crucial to avoid leakage to minimize the chances of recurrence [12]. Imaging methods such as radiology, ultrasonography, computed tomography (CT), and magnetic resonance imaging (MRI) are crucial for final diagnosis [13]. Chemotherapy utilizing benzimidazole compounds such as albendazole and mebendazole represents a common practice, with albendazole demonstrating efficacy in diminishing cyst size and quantity. Although adverse reactions like nausea, hepatotoxicity, neutropenia, and alopecia have been associated with albendazole use, continuous treatment has exhibited promising outcomes with manageable side effects [14]. The suggestion that long‐term albendazole (ABZ) treatment may serve as a safe and effective therapeutic approach for individuals with disseminated abdominal cystic echinococcosis highlights the importance of clinical trials to outline the optimal management strategies for this parasitic infection [15].

Estimations indicate a predominant global prevalence of individuals harboring hydatid cysts, with the potential to result in prominent morbidity and mortality in the absence of timely intervention [16].

## 8 Conclusion

This case highlights the challenges in diagnosing ovarian hydatid cysts, which can be mistaken for other ovarian conditions. Early detection is crucial, especially in endemic regions, for accurate management. Imaging tests aid in diagnosis, but histopathological examination confirms it. Surgery and anthelmintic therapy are key for treatment, reducing recurrence risks. This highlights the need to consider hydatid disease in ovarian cyst diagnoses, even without prior history. A multidisciplinary approach is essential for effective management of this rare condition.

## Author Contributions

**Fatemeh Asadi Kohbad:** conceptualization. **Razieh Akbari:** supervision. **Maryam Forouzin:** writing – original draft. **Fatemeh Nili:** investigation, methodology. **Leila Asadi:** writing – review and editing. **Marjan Ghaemi:** investigation.

## Funding

The authors have nothing to report.

## Consent

Informed written and verbal consent was obtained from the patient for the publication of this study.

## Conflicts of Interest

The authors declare no conflicts of interest.

## Data Availability

Data is available upon request.
